# Progress with the Second Dose Measles Vaccine Introduction and Coverage in the WHO African Region

**DOI:** 10.3390/vaccines12091069

**Published:** 2024-09-18

**Authors:** Balcha G. Masresha, Messeret E. Shibeshi, Gavin B. Grant, Cynthia Hatcher, Charles S. Wiysonge

**Affiliations:** 1 Vaccine Preventable Diseases Program, World Health Organization, Regional Office for Africa, Brazzaville P.O. Box 06, Congo; sheyc@who.int; 2 Vaccine Preventable Diseases Program, World Health Organization, Regional Office for Africa, Inter-Country Support Team, Harare P.O. Box CY 348, Zimbabwe; eshetum@who.int; 3 Global Immunization Division, Global Health Center, U.S. Centers for Disease Control and Prevention, Atlanta, GA 30333, USA; gig9@cdc.gov (G.B.G.); ctp0@cdc.gov (C.H.)

**Keywords:** measles, elimination, measles second dose, routine immunization, second-year of life, Africa

## Abstract

Introduction: To achieve global and regional measles elimination objectives, the World Health Organization (WHO) recommends coverage of 95% or higher with two doses of measles-containing vaccine. A second dose of measles-containing vaccine (MCV) is typically administered in the second year of life after 12 months of age. Methods: We reviewed WHO-UNICEF estimates of national coverage (WUENIC) for the first and second doses of MCV (MCV1 and MCV2, respectively) and calculated drop-out rates between MCV1 and MCV2 for countries in the WHO African Region. Results: From 2013 to 2023, estimated regional MCV2 coverage increased from 7% to 49%, and at the end of 2023, 43 (91%) countries had introduced MCV2 into their routine immunization programs. Countries with more antigens provided in the second year of life had higher mean and median MCV2 coverage levels, and lower drop-out rates between MCV1 and MCV2, as compared to countries providing only MCV2. Discussion: Despite substantial progress, MCV2 coverage remains below the required levels to achieve and sustain elimination, and many countries have high drop-out rates between MCV1 and MCV2 coverage, indicating challenges in reaching children over 12 months of age. Increasing coverage of MCV2 and other vaccines in the second year of life is essential to achieving higher and equitable routine immunization coverage. This will require continued efforts to understand and mitigate barriers to reaching children after 12 months of age and accelerated implementation of available tools.

## 1. Introduction

The African Regional Immunisation Strategic Plan 2021–2030 aims to achieve measles and rubella elimination in at least 80% of the countries by 2030 [1]. To achieve this goal, the World Health Organization (WHO) recommends reaching all susceptible persons with two doses of measles-containing vaccine (MCV) [2]. The routine administration of a second dose of measles-containing vaccine (MCV2), usually in the second year of life (2YL) between 15 and 18 months of age, reduces susceptibility in up to 15% of individuals who do not seroconvert after the first dose of MCV (MCV1) at 9 months of age [2]. MCV2 was originally recommended for countries with MCV1 coverage at or above 80%, but WHO guidance was updated in 2017 to recommend MCV2 introduction, regardless of MCV1 coverage [2]. In addition to MCV2, the 2YL platform provides a scheduled vaccination opportunity to administer MCV1 to unvaccinated children to reach the high population immunity levels needed to prevent measles transmission and integrate with other immunizations and health services [3].

One of the Immunization Agenda 2030 (IA2030) strategic priorities is for all people to benefit from recommended immunizations throughout the life course, effectively integrated with other essential health services [1,4]. MCV2 coverage is one of the critical indicators used to monitor progress towards the Sustainable Development Goals (SDGs) [4]. As of 2023, 190 (98%) of 194 member states have introduced MCV2, and the estimated global MCV2 coverage was 74% [5]. However, an estimated 11 million children did not receive MCV2 in routine immunization in 2022, and global and regional MCV2 coverage remains below the target of 95% or higher [6]. 

Operational guidelines are available to assist countries to plan and prepare for MCV2 introduction, and countries are encouraged to conduct post-introduction evaluation (PIE) exercises within the first few months of new vaccine introduction to assess roll-out and address any gaps as early as possible [7] [AFRO guidelines]. 

This paper describes the progress in introducing a second dose of measles vaccination in the WHO African Region (AFR) more than a decade after the global push for introduction with support from Gavi, the Vaccine Alliance (Gavi). We reviewed the vaccination coverage levels attained within the context of the broader vaccination initiative in the second year of life platform. 

## 2. Methods

Every year, all countries report to WHO and UNICEF their national MCV1 and MCV2 administrative coverage (as calculated by dividing the total number of doses administered to children by the number of children in the respective target age groups), as well as the national vaccination schedule. WHO and UNICEF generate estimates of coverage for key antigens, including first and second doses of measles-containing vaccines, based on the reported data, as well as national coverage estimates, data from vaccination coverage surveys, and other sources of data validation [5,8]. For the status regarding the introduction of MCV2, we reviewed the program information as of the end of 2023. Using the officially published WHO-UNICEF estimates of national immunization coverage (WUENIC) for MCV1 and MCV2, we examined the MCV2 coverage rates over the ten-year period from 2013 to the latest available coverage estimate data from 2023. For the year 2023, using the WUENIC estimates, we calculated the drop-out rates between MCV1 and MCV2 and the ratio of MCV2 coverage to the MCV1 coverage for the countries in the AFR region that have had MCV2 in their routine immunization schedules for at least three years. To evaluate the role of the health system strength in countries introduced prior to 2022, we correlated MCV2 coverage to the universal health coverage (UHC) service index and general domestic general health expenditures (GGHEs) (as a percent of Gross Domestic Product) [9].

## 3. Results

MCV2 introduction in AFR has accelerated from 2013, when only 11 (24%) of 46 countries had introduced MCV2, to 43 (92%) of 47 countries having introduced MCV2 by the end of 2023. At the end of 2023, only Benin, Central African Republic (CAR), Gabon, and South Sudan had not introduced MCV2.

Nearly all AFR countries schedule the MCV2 dose at 15 or 18 months, except for South Africa, which provides MCV2 at 12 months of age, Mauritius at 17 months of age, and Cape Verde and Seychelles at 6 years of age. Mauritius only shifted to 17 months of age from 5 years of age in 2023 to ensure the early protection of children. 

**Figure 1:**
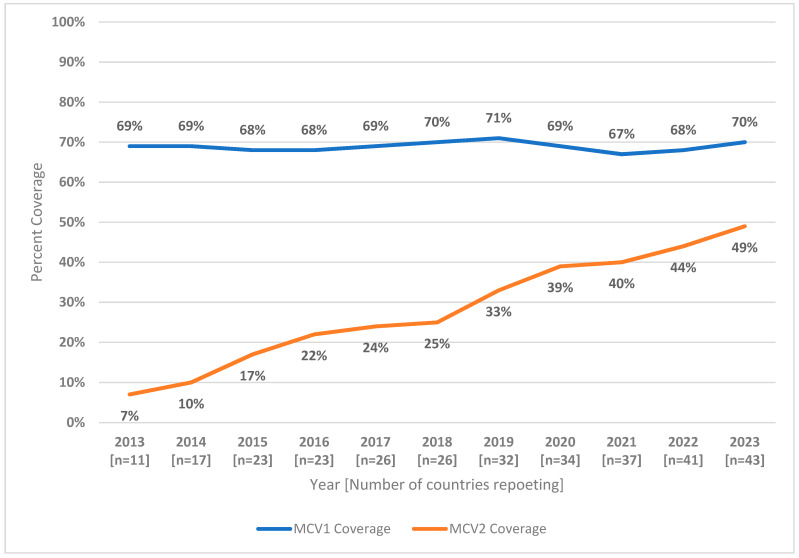
MCV1 and MCV2 estimated coverage (WUENIC estimates)—World Health Organization African Region, 2013–2023. Abbreviations: MCV1 = first dose of measles-containing vaccine; MCV2 = second dose of measles-containing vaccine; WUENIC = WHO and UNICEF estimates of national immunization coverage.

The MCV1 and MCV2 WUENIC coverage estimates for AFR from 2013 to 2023 are given in Figure 1. As of December 2023, the estimated regional MCV2 coverage reached 49%, a substantial increase from the 7% coverage documented in 2013. Only three countries had ≥90% MCV2 coverage in 2023: Algeria (93%), Mauritania (94%), and Sao Tome and Principe (90%). Eleven countries had MCV2 coverage levels of ≥80%, while 14 countries had coverage of less than 50% (Angola, Cameroon, Central African Republic, Chad, Republic of Congo, Cote d’Ivoire, DR Congo, Equatorial Guinea, Gabon, Guinea, Guinea-Bissau, Madagascar, Mauritania, Mozambique, Nigeria, South Sudan, and Uganda) (Table 1). 

Among the 15 countries that have had MCV2 in their routine program for at least ten years, the mean MCV1 and MCV2 coverage levels were 90% and 83%, and the mean and median drop-out rates between MCV1 and MCV2 coverage were both 8%. In 17 countries with MCV2 in their program for between 5 and 9 years, the mean drop-out rate was 16%, with a median of 15.5%. In seven countries where MCV2 was introduced only 3–5 years ago, the average and median drop-out rate was 26% and 23% (Table 1).

As of 2022, a total of 19 countries have reported providing antigens other than MCV2 in the 2YL platform in their routine immunization programs (Table 2). Of the 19, ten provide either pentavalent, diphtheria, tetanus, and pertussis (DTP), or tetanus toxoid and diphtheria (DT) vaccine; nine provide oral polio (OPV) or inactivated polio (IPV), five meningococcal A conjugate vaccine, and two provide malaria vaccine. Higher MCV2 coverage was associated with more vaccines provided during 2YL; countries with 0, 1, or 2 additional vaccines showed an increasing MCV2 coverage (39%, 61%, and 92%, respectively) and a decreasing drop-out rate of 34%, 26%, and 11%, respectively.

The correlation between MCV2 coverage levels and health system indicators demonstrated a positive relationship, with a moderate association of 0.52 with General Governmental Health Expenditures per capita (GGHE) and 0.59 for Universal Health Coverage Service Index (UHC) (see Supplementary Figure S1).

## 4. Discussion

Following the change in the WHO policy recommendations in 2017 to include a second dose in all countries independent of first dose coverage and the availability of funding support from Gavi for introduction, there has been significant progress in MCV2 introduction from 2013 to 2022 in AFR. However, in 2023, the regional MCV2 coverage remains low at 49% compared with global MCV2 coverage of 74% [5,6], both of which are inadequate for achieving and sustaining the elimination of measles. Most countries have failed to raise their MCV2 coverage levels near MCV1 coverage levels, indicating that a large proportion of children drop out between the first and second doses. The declines in vaccination coverage during the COVID-19 pandemic have left more children unprotected against measles in 2022, risking large and disruptive outbreaks in some countries. In this study, we note that despite the trend showing an overall increase in regional-level MCV2 coverage in the past few years, the number of countries meeting the 95% target for MCV2 coverage declined in 2022, an impact of the COVID-19 pandemic [6,10]. The milestones to reaching measles elimination in AFR include the attainment of 90% coverage with MCV2 in 40% of countries by 2025 and in all countries by 2030; these will not be met unless progress is accelerated [1]. 

The fact that many countries continue to have low MCV2 coverage and high drop-out rates more than three years post-introduction is evidence that challenges remain with the 2YL platform. Despite the availability of initial one-time Gavi funding to support the operational costs related to introducing MCV2 in eligible countries, the preparation for MCV2 introduction in many countries has been sub-standard. Post-introduction evaluation (PIE) exercises have documented that MCV2 introductions in some countries were not implemented with meticulous logistics, social mobilization and communications, micro-planning, health worker training, revision of monitoring tools, conducting caretaker awareness for the new point of health service contact, or putting in place means of monitoring [11]. At least seven of the countries (Chad, Congo, Equatorial Guinea, Guinea, Guinea Bissau, Madagascar, and Mauritania) that introduced MCV2 since 2019 have not yet implemented comprehensive PIE exercises, which limit their ability to identify and address structural barriers in a timely manner and develop plans to increase MCV2 coverage. 

A global research prioritization exercise in 2016 identified that there is a need to determine the effectiveness of strategies to increase coverage with MCV1 and MCV2, such as strategies to (1) reduce missed opportunities for vaccination (MOV), (2) improve defaulter tracing, (3) improve coverage through use of a five-dose measles vial, and (4) improve coverage using school-based platforms for the checking of vaccination status and the delivery of catch-up doses [12].

To date, evidence has been generated surrounding the effectiveness of some of these strategies. One documented barrier to increasing measles vaccination coverage has been the concern about vaccine wastage in countries using 10-dose vials, where health workers open vials only when they are certain they can utilize the majority of the doses within six hours once reconstituted. A study in Nigeria showed that vaccinators expect a minimum of six children to be present prior to opening a 10-dose MCV vial [13]. Similarly, many health facilities do not provide daily measles vaccination services, resulting in missed opportunities to deliver MVC2 [14]. 

As of the end of 2023, a total of 12 countries (Botswana, Cape Verde, Comoros, DR Congo, Equatorial Guinea, Eritrea, Eswatini, Lesotho, Niger, Mauritius, Seychelles, and Zimbabwe) are using five-dose or lesser-dose vials instead of the 10-dose measles-containing vaccine vials. Studies have shown that the introduction of MCV in five-dose vials leads to better health worker readiness to open vials, even with fewer eligible children at service delivery sites, and have demonstrated a statistically significant increase in both MCV1 and MCV2 coverage compared with districts using 10-dose vials [15,16]. 

Missed opportunities for vaccination (MOV) persist in many countries, and in one study, among children who were due for at least one vaccination dose, 76% had at least one MOV [14,17]. Many studies have documented healthcare workers’ reluctance or refusal to open a multi-dose vial unless a critical number of children is gathered, especially for lyophilized vaccines such as BCG, MCV, measles-rubella (MR), and Yellow Fever (YF) vaccines [13,14,15,18]. Many countries have adopted school-based vaccination to reach older children with missed doses [19]. On the caregiver side, a study in Ghana found that the caregivers’ awareness of the routine immunization schedule was an important determinant of childhood vaccination uptake of MCV2 and meningitis A (MenA) conjugate vaccine [20]. Other factors like maternal age, rural residency, and birth order also affected the uptake of the vaccines in the 2YL [11,20,21]. Vaccine hesitancy may also be a factor in stagnating coverage, but the impact specifically on MCV2 and other 2YL antigens has not been well described [22].

There is a moderate correlation between health system strength indicators and MCV2 coverage; both coverage and government spending on the healthcare, but are not sufficient to determine coverage, but do continue to emphasize that the degree of health care financing and universal health coverage contributes to high MCV2 coverage levels. As a caveat, these indicators do not measure the quality of services provided, nor do they reflect demand-side factors (e.g., desire for a second measles vaccination dose).

A robust 2YL platform is essential to ensure that young children are fully protected against measles and against other pathogens targeted by vaccines such as meningococcal A conjugate vaccine (Men A), a booster dose of diphtheria-tetanus-pertussis (DTP)-containing vaccine, and additional doses of pentavalent and polio vaccines. Only 19 (40%) of 47 AFR countries include DPT4 or other vaccines in the second year of life in addition to MCV2. The strengthening of the 2YL platform can be a critical point of entry for catch-up vaccination to reach children who may have missed primary doses in infancy. One analysis in South Africa found that the 2YL visit increased MCV coverage by 3%, but only 20% of children missing a pentavalent dose received a dose during this visit [23]. In addition to the 2YL platform, nine (19%) countries have scheduled vaccination appointments at the age of 5 or 6 years, coinciding with school entry. Advancing school health programs and policies requiring proof of vaccination upon school entry would help to advocate for and ensure complete measles vaccination.

The recent WHO prequalification of malaria vaccines with high national demand creates an opportunity to leverage and strengthen the 2YL platform, as many countries opt to give the fourth dose of malaria vaccine in the second year of life using established vaccination contact points [24]. Assessing the impact of malaria vaccination on increasing MCV2 and other antigens provided during the 2YL will be important to guide programs to refine vaccination schedules and service delivery approaches. 

Countries are encouraged to implement detailed subnational level analysis of immunization program performance, including the 2YL platform, and as needed, conduct program reviews (either desk reviews or extensive field level reviews) and implement post-introduction evaluation to monitor the health of the 2YL and address policy and program gaps. In addition, it is important to remove any policy barriers to vaccinating eligible unvaccinated children at any age, including putting in place robust screening of vaccination status at school entry [25]. 

This study has several limitations. The national coverage estimates do not reflect the significant coverage diversity at the subnational level, e.g., among provinces and districts, especially in large countries [26]. In addition, data quality limitations may result in inaccurate estimates of coverage in some countries. A thorough understanding of all challenges to MCV2 service delivery, including the role of vaccine demand and quality of vaccine introduction activities, was beyond the scope of this analysis, though it likely contributes to the current challenges with improving coverage. 

## 5. Conclusions

Increasing coverage of MCV2 and other vaccines in the second year of life is essential to achieving the African Regional Immunization Strategic Plan 2021–2030 and the Immunization Agenda 2030 objectives to eliminate measles and protect children from illness, disability, and death. Significant progress has been made in the African region to increase both coverage, and the number of countries offering MCV2, but no country has achieved the goal of ≥95% coverage, and only three (7%) countries have a coverage of ≥ 90%. Global commitment to immunization and other essential health interventions across the life course underscores the importance of strengthening the 2YL platform, increasing the utilization of five-dose vials and developing locally tailored strategies to reach children after 12 months of age, which are essential to achieving these objectives.

## Figures and Tables

**Table 1:** MCV1 and MCV2 estimated coverage (WUENIC estimates), drop-out rates, vaccine type and year of MCV2 introduction—World Health Organization African Region, 2022.

Country	Type of Vaccine Used	Year MCV2 Introduced	MCV1 Coverage (WUENIC 2023)	MCV2 Coverage (WUENIC 2023)	Drop-Out Rate ^1^
Algeria	MMR	Pre-2000	99	93	6%
Angola	MR	2015	50	35	30%
Benin	M	N/A	52	0	N/A
Botswana	MR	2011	97	79	19%
Burkina Faso	MR	2014	94	71	24%
Burundi	MR	2013	86	80	7%
Cabo Verde	MMR	2010	95	86	9%
Cameroon	MR	2019	71	45	37%
Central African Republic	M	N/A	41	0	N/A
Chad	M	2022	63	35	44%
Comoros	MR	2021	70	79	−13%
Congo	MR	2019	65	34	48%
Côte d’Ivoire	MR	2021	70	43	39%
DR Congo	M	2022	52	18	65%
Equatorial Guinea	M	2021	61	28	54%
Eritrea	MR	2012	93	85	9%
Eswatini	MR	Pre-2000	85	82	4%
Ethiopia	M	2019	61	53	13%
Gabon	M	N/A	66	0	N/A
Gambia	MR	2012	80	73	9%
Ghana	MR	2012	90	78	13%
Guinea	M	2022	47	26	45%
Guinea-Bissau	M	2022	72	36	50%
Kenya	MR	2013	91	76	16%
Lesotho	MR	Pre-2000	90	82	9%
Liberia	M	2019	82	60	27%
Madagascar	M	2020	51	47	8%
Malawi	MR	2015	87	65	25%
Mali	MR	2019	73	59	19%
Mauritania	MR	2023	92	24	74%
Mauritius	MMR	2003	96	94	2%
Mozambique	MR	2015	65	44	32%
Namibia	MR	2017	86	62	28%
Niger	M	2014	80	68	15%
Nigeria	M	2019–2020	60	38	37%
Rwanda	MR	2014	96	88	8%
Sao Tome and Principe	MR	2013	86	90	−5%
Senegal	MR	2014	76	64	16%
Seychelles	MMR	Pre-2000	93	89	4%
Sierra Leone	MR	2015	90	73	19%
South Africa	M	Pre-2000	80	84	−5%
South Sudan	M	N/A	72	0	N/A
Togo	MR	2019	72	58	19%
Uganda	MR	2022	93	21	77%
United Republic of Tanzania	MR	2014	91	78	14%
Zambia	MR	2013	90	75	17%
Zimbabwe	MR	2015	90	77	14%

Abbreviations: M = measles vaccine; MCV1 = first dose of measles-containing vaccine; MCV2 = second dose of measles-containing vaccine; MR = measles and rubella vaccine; MMR = measles, mumps, and rubella vaccine; N/A = not applicable; WUENIC = WHO and UNICEF estimates of national immunization coverage. ^1^ MCV1 − MCV2 drop-out rate = (MCV1 − MCV2)/MCV1 coverage.

**Table 2:** Vaccines other than MCV2 offered after 12 months of age to pre-school children. World Health Organization African Region, 2022. Official country reports through the WHO-UNICEF Joint Reporting Form (JRF).

Country	Other Vaccines Provided in the 2nd Year of Life	Other Vaccines Provided by Age of School Entry
Algeria	None	OPV, DT and MR2 at 6 years
Angola	None	None
Benin	None	None
Botswana	OPV and DT at 18 months	DT at 7 years
Burkina Faso	Meningitis Conjugate Vaccine-4, MenA	None
Burundi	DTP4	None
Cabo Verde	Penta4, OPV	None
Cameroon	None	None
Central African Republic	None	None
Chad	None	None
Comoros	None	None
Congo	None	None
Côte d’Ivoire	None	None
DR Congo	None	None
Equatorial Guinea	Penta4 at 18 months	None
Eritrea	Men-A	None
Eswatini	OPV at 18 months	OPV and DT at 5 years
Ethiopia	None	None
Gabon	None	None
Gambia	DTP4 and OPV4 at 18 months	None
Ghana	RTS,S and Men A at 18 months	None
Guinea	Men A at 15 months	None
Guinea-Bissau	None	None
Kenya	None	None
Lesotho	DT at 18 months	Td at 6 years
Liberia	None	None
Madagascar	None	None
Malawi	RTS,S4 at 22 months	None
Mali	None	None
Mauritania	None	None
Mauritius	Penta4 and IPV at 18 months	Tdap and IPV at 5 years
Mozambique	None	Td at 6 years
Namibia	None	OPV and DT at 5 years
Niger	None	None
Nigeria	None	None
Rwanda	None	None
Sao Tome and Principe	OPV4 at 15 months	None
Senegal	None	None
Seychelles	DPT4 and OPV4 at 18 months	DT and OPV at 6 years
Sierra Leone	None	None
South Africa	Penta4 and IPV at 18 months	Td at 6 years
South Sudan	None	None
Togo	Men A at 15 months	None
Uganda	None	None
United Republic of Tanzania	None	None
Zambia	OCV at 13 months	None
Zimbabwe	DTP4 and OPV at 18 months	None

Abbreviations: DT = tetanus toxoid and diphtheria vaccine, children’s dose; DTP4 = fourth dose of diphtheria, tetanus, and pertussis vaccine; IPV = inactivated polio vaccine; MenA = meningococcal A conjugate vaccine; MCV2 = second dose of measles-containing vaccine; MR2 = second dose of measles and rubella vaccine; OCV = oral cholera vaccine; OPV = oral polio vaccine; OPV4 = fourth dose of oral polio vaccine; Penta4 = fourth dose of pentavalent vaccine; RTS,S = RTS,S/AS01 malaria vaccine; RTS,S4 = fourth dose of malaria vaccine; Td = tetanus toxoid and diphtheria vaccine, older children and adult dose; Tdap = tetanus, diphtheria, and acellular pertussis vaccine.

## Data Availability

Sources for vaccination coverage data presented in this analysis were cited.

## Supplementary Materials

The following supporting information can be downloaded at https://www.mdpi.com/article/10.3390/vaccines12091069/s1, Figure S1. Association of a second dose of measles-containing vaccine (MCV2) and Universal Health Coverage Service Index (UHC) and General Governmental Health Expenditures per capita (in US dollars) for countries in the African Region that had been introduced prior to 2022.
