# Supplementary material for: Progress with the Second Dose Measles Vaccine Introduction and Coverage in the WHO African Region
Source: Vaccines (Basel). 2024 Sep 18;12(9):1069. doi: 10.3390/vaccines12091069 (PMC11435470; doi:10.3390/vaccines12091069)
Supplement: Supplementary file 1 [file vaccines-12-01069-s001.zip › vaccines-3126068-supplementary.pdf]

Supplementary Material

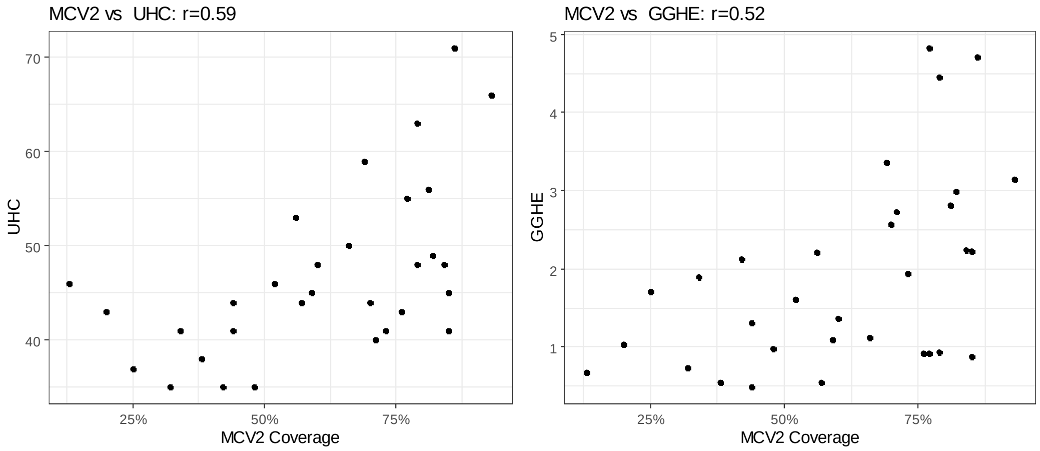

Figure S1. Association of a second dose of measles-containing vaccine (MCV2) and Universal Health Coverage Service Index (UHC) and General Governmental Health Expenditures per capita (in US dollars) for countries in the African Region that had been introduced prior to 2022.
